# Supplementary material for: ‘They Are Kids, Let Them Eat’: A Qualitative Investigation into the Parental Beliefs and Practices of Providing a Healthy Diet for Young Children among a Culturally Diverse and Deprived Population in the UK
Source: Int J Environ Res Public Health. 2021 Dec 11;18(24):13087. doi: 10.3390/ijerph182413087 (PMC8700847; doi:10.3390/ijerph182413087)
Supplement: Supplementary file 1 [file ijerph-18-13087-s001.zip › ijerph-1437098-supplementary.pdf]

Supplementary File S1: Topic Guide Questions

I would like to ask you a little bit about feeding your child from birth through to weaning.

- *What feeding method did you choose for your new-born baby?*
  - *Probe: whether they breast fed and or/formula, exclusively breast feeding or combined, expressed the breast milk, duration.*
  - *Probe: child's response to feeding method.*
- *Who/what influenced your decisions about feeding your child/children?*
  - *Probe: for breast feeding vs. formula feeding, family, friends, internet, TV, radio, midwives, health visitors, cost, availability, culture and religion.*
- *What was your (and your wife's/partner's) experience of feeding your child/children?*
  - *Probe: for breast feeding vs. formula feeding, difficulties and easy/good. If received support/advice or not, who was support/advice from.*
- *What services/interventions did you access to support you and what were your experiences of accessing these*
  - *Probe: Breastfeeding clinics, healthcare professionals, other*
